# High Cholesterol Diet-Induced Changes in Oxysterol and Scavenger Receptor Levels in Heart Tissue

**DOI:** 10.1155/2018/8520746

**Published:** 2018-06-13

**Authors:** Erdi Sozen, Burak Yazgan, Ali Sahin, Umit Ince, Nesrin Kartal Ozer

**Affiliations:** ^1^Department of Biochemistry, Faculty of Medicine, Genetic and Metabolic Diseases Research and Investigation Center (GEMHAM), Marmara University, 34854 Maltepe, Istanbul, Turkey; ^2^Central Research Laboratory, Amasya University, 05100 Amasya, Turkey; ^3^Acıbadem University and Acıbadem Health Group, Istanbul, Turkey

## Abstract

Involvement of high cholesterol and oxidative stress in cardiovascular diseases is well studied, as it can be hypothesized that various products originated from lipid peroxidation, such as oxysterols, or affected protein expression might lead to cardiomyocyte damage followed by the pathological modifications. Although oxidation of excessive cholesterol to oxysterols in elevated stress conditions is identified by a number of studies, the role of a high cholesterol diet in regulating fatty acid and oxysterol accumulation, together with scavenger receptor mRNA levels, in the heart remains little investigated. Our study provides a detailed analysis of the changes in fatty acid, oxysterol, and scavenger receptor profiles and its relation with histological alterations in the heart tissue. We evaluated alterations of fatty acid composition, by the GC-MS method, while 4*β*-, 25-, and 27-hydroxycholesterol and 7-ketocholesterol levels by means of LC-MS/MS in high cholesterol diet-fed rabbits. Additionally, a number of proteins related to lipid metabolism and scavenger receptor mRNA expressions were evaluated by Western blotting and RT-PCR. According to our *in vivo* results, a high cholesterol diet enhances a number of unsaturated fatty acids, oxysterols, and LXR*α*, in addition to CD36, CD68, CD204, and SR-F1 expressions while *α*-tocopherol supplementation decreases LXR*α* and SR expressions together with an increase in 27-hydroxycholesterol and ABCA1 levels. Our results indicated that the high cholesterol diet modulates proteins related to lipid metabolism, which might result in the malfunction of the heart and *α*-tocopherol shows its beneficial effects. We believe that this work will lead the generation of different theories in the development of heart diseases.

## 1. Introduction

Cholesterol is a key component in regulating various cell functions, including the permeability and fluidity of membrane, steroid hormone synthesis, and bile acids. After the delivery of cholesterol into the cell via lipoprotein or scavenger receptors (SRs), it might be tranferred to (i) endoplasmic reticulum (ER), inducing the sequestration of sterol regulatory element-binding proteins (SREBPs) to reduce the synthesis and uptake of cholesterol, (ii) plasma membrane, enhancing the efflux of cholesterol (known as reverse cholesterol transport (RCT)) through ATP-binding cassette transporter A1 (ABCA1), and (iii) mitochondrial sterol 27-hydroxylase (CYP27A1), increasing endogenous production of 27-hydroxycholesterol followed by the activation of liver X receptors (LXRs) [1].

Increased production of reactive oxygen species (ROS), known as oxidative stress, is enhanced by the imbalance between antioxidant systems and cellular ROS production. Generation of oxygenated cholesterol products (oxysterols) inside the cell is majorly produced either as a result of the free radical attacks or enzymatic reactions and known as an essential reaction in cholesterol-mediated tissue damage [2]. For instance, while unoxidized cholesterol does not contain any inflammatory effect, endogenously originated oxysterols have shown to enhance inflammation in vascular remodeling [3]. Of the oxysterols, 7-ketocholesterol is one of the major oxysterols found increased in the plasma of patients with coronary artery disease [4]. 7-Ketocholesterol also involves in atherosclerosis development either by inducing apoptosis [5] or by inhibiting reverse cholesterol transport [6].

Elevated levels of oxysterols have been also indicated as a regulator of gene transcription. LXR*α* and PPAR*α* are two transcription factors that are highly expressed in macrophages and regulate the transcription of genes in modulating lipid metabolism, inflammation, and cholesterol efflux [7, 8]. Related studies have identified LXR*α* as a sterol receptor that modulates the expression of lipid metabolism-related genes, in addition to the reduction of cholesterol accumulation by upregulating ABCA1-related cholesterol efflux, following the binding of 25- and 27-hydroxycholesterol [8–10]. Oxysterols might also facilitate SREBPs that reduce the cholesterol uptake and synthesis [11].

SRs are identified as the members of the “membrane-bound receptor” family that can specifically bind to various ligands such as oxidized phospholipids/lipoproteins, modified lipid particles, and pathogens. Based on the current understanding of their structure and biological function, SRs have been classified into classes A–J [12]. Due to their variety on ligand binding and signal transduction, SRs are identified not only in atherosclerosis development but also in the immune response, inflammation, and neurodegenerative diseases. For instance, while oxLDL binding to class B scavenger receptor SR-B2 (CD36) activates signaling mechanisms including JNK, p38 MAPK, and tyrosine kinase Fyn [13], class D scavenger receptor SR-D1 (CD68) can recognize lectins, selectins, and OxLDL followed by the regulation of phagocytosis [14, 15].

However, the particular mechanisms of lipid and oxysterol metabolism, together with SR expressions, in heart tissue following a high cholesterol diet and *α*-tocopherol supplementation have not yet been fully clarified. The aim of our study is to investigate the alterations of lipid metabolism and SR expression in a hypercholesterolemic rabbit model and whether these alterations are affected by the supplementation of *α*-tocopherol, the most active form of vitamin E. To do that, intracellular accumulation of fatty acids and various oxysterols containing 4*β*-, 25-, and 27-hydroxycholesterol and 7-ketocholesterol, in addition to SREBP1c, LXR*α* and PPAR*α* levels, was investigated in the heart tissue. Moreover, SR (CD36, CD68, CD204 (SR-A), SR-B1, SR-F1, and SR-G) and RCT system (ABCA1) were evaluated by measuring mRNA expressions. We observed high cholesterol diet-induced levels of a number of unsaturated fatty acids, oxysterols, and LXR*α*, in addition to CD36, CD68, CD204, and SR-F1 expressions. In this context, *α*-tocopherol supplementation showed its beneficial effect by decreasing LXR*α* and SR expressions while enhancing 27-hydroxycholesterol and ABCA1 levels.

## 2. Methods

### 2.1. Animals and Diets

All experimental procedures were approved by the Marmara University Ethics Committee, Istanbul (protocol number 872010). Twenty male albino rabbits (2-3 months old) were divided randomly into four groups which were fed with 100 g per day of vitamin E poor diet. The first group was only fed with an *α*-tocopherol poor diet. The second group was fed with the *α*-tocopherol poor diet containing 2% cholesterol. The third group was fed with the *α*-tocopherol poor diet containing 2% cholesterol with daily intramuscular injections of *α*-tocopherol (50 mg/kg), and the fourth group was fed with the *α*-tocopherol poor diet with daily intramuscular injections of *α*-tocopherol (50 mg/kg). *α*-Tocopherol concentration was in accordance with the previous literature [16, 17].

After 8 weeks of feeding, following overnight fasting, rabbits were anesthetised using 50 mg/kg ketamine hydrochloride and 5 mg/kg xylazine hydrochloride. The blood was taken for cholesterol and *α*-tocopherol measurements. The heart tissues of each animal were removed, rapid-frozen in liquid nitrogen, and stored at −80°C for GC-MS, LC-MS/MS, qPCR, and immunoblotting experiments.

### 2.2. Measurement of Cholesterol and *α*-Tocopherol Levels in Serum

Serum cholesterol levels were determined using an automated enzymatic technique by Hitachi Modular system P800 (Roche). The levels of alpha-tocopherol were determined in serum samples by using reversed-phase high-performance liquid chromatography (HPLC) according to Nierenberg and Nann [18]. Briefly, samples were dissolved in ethanol and applied to a C18 column (5 *μ*m, 4.6 × 250 mm). MeOH : dH_2_O (95 : 5, *v*/*v*) was used as a mobile phase, and detections were performed by a UV detector (Thermo) at 294 nm. The relative standard peak areas of *α*-tocopherol were determined and compared with peak areas of samples to calculate concentration as *μ*g/mL.

### 2.3. Measurement of *α*-Tocopherol in Heart Tissue

Two mL of hexane was added into a clean test tube with 100 mg of heart tissue and incubated at 70 C for 10 min followed by the homogenization using an IKA Ultraturrax homogenizator at 15.000 rpm for 30 seconds. During the incubation, the test tube was vortexed every 5 min. The hexane layer was transferred to a clean tube after it was separated by the field of hypercholesterolemia mediated heart disease aging at 1000 ×g for 10 min Another 2 mL of hexane was added and vortexed for 5 min, with the residual aqueous layer. The test tube was centrifuged again. The separated hexane layer was combined with the previous one. Following the evaporation under nitrogen, ethanol was used to dissolve the sample and *α*-tocopherol was analyzed by LC-MS/MS.

LC-MS/MS analyses were performed by reversed phase HPLC on an Inertsil ODS-3 column (10 cm × 3 mm i.d., 3 *μ*m particle size) using a Shimadzu UPLC system. The mobile phase was as follows: solvent A, distilled water containing 0.1% formic acid, and solvent B, methanol containing 0.1% formic acid. A constant flow rate of 0.5 mL/min and a gradient profile from 80% to 100% of solvent B were employed. Detection was done with an Applied Biosystems Sciex API 4000 QTrap mass spectrometer (Applied Biosystems) equipped with a TurboIonSpray ionization source. Data were acquired in the selective positive multiple reaction monitoring mode (MRM) alternating the following transitions: [*M* + *H*] + = 431.5 to 165.0 and 431.5 to 137.0.

### 2.4. Measurement of MDA in Heart Tissue

To prepare a MDA standard solution, 20 mL of 0.1 M HCl was added to 34 *μ*L of 1,1,3,3-tetramethoxypropane (TMP) followed by incubation at 40°C for 1 h to hydrolyse TMP into MDA. The concentration of MDA in the standard solution was determined by measuring its absorbance at 245 nm (*ε* = 13,700) and freshly diluted with deionized water to establish a calibration curve.

Tissue samples (100 mg) were homogenized in 0.1 M phosphate buffer (pH 7.4) followed by an addition of 1 M KOH and 0.02 M BHT. After then, they were left to incubation at 60°C for 1 h with continuous shaking at dark. Following acidification with concentrated HCl to pH 2, they were centrifuged at 15000 ×g for 5 min at 4°C. The resulting supernatants were then derivatized with an equal volume of DNPH (1.2 mM) at 50°C for 60 min and protected from light. After derivatization, the sample was allowed to cool down and centrifuged at 15,000 ×g for 7 min at 4°C. The supernatant was transferred to a clean vial, filtered by 45 *μ*m filter, and 20 *μ*L of a resulting solution was injected onto the LC-MS/MS instrument (Shimadzu UPLC, AB-Sciex 4000 QTrap) for chromatographic analysis. The relative standard peak areas of MDA-DNPH were determined and compared with the peak areas of samples to calculate concentration as ng/mL.

### 2.5. Protein Carbonyl Analysis in Heart Tissue

Following the electroblotting step, the membranes were kept in TBS (100 mM Tris, 150 mM NaCl, pH 7.5) containing 20% methanol for 5 min, washed in 2 N HCl for 5 min, incubated with 10 mM DNPH solution for 5 min, washed 3 × 5 min in 2 N HCl, and washed 5 × 5 min in 50% methanol. A DNPH-treated membrane was blocked with 5% nonfat dry milk in TBST for 1 h at room temperature followed by the blocking step. A blocked membrane was incubated with anti-DNP antibody (Sigma) in 5% nonfat dry milk/TBST for 1 h at room temperature with constant agitation. A blotted membrane was washed 3 × 5 min with TBST and incubated with HRP-conjugated secondary antibody followed by the washing step for 5 × 5 min with TBST. The membrane was developed using a chemiluminescence kit (Cell Signaling), and blots were quantified/normalized with *β*-actin by densitometry using Image J software.

### 2.6. Light Microscopy Examination of Heart Tissue

Heart tissue samples were fixed in 10% buffered formaldehyde for 4 hours, dehydrated, and incubated in xylol for 1 hour twice, embedded in paraffin, and sectioned in 5 *μ*m thickness onto glass slides. Hematoxylin-eosin and Masson's trichrome stainings were performed and examined under light microscopy (Leica) at 200x magnification to evaluate the pathological features.

### 2.7. Determination of Fatty Acid Profile by GC-MS in Heart Tissue

Heart tissue fatty acids were extracted according to the Bligh and Dyer method [19]. To determine the fatty acid composition of extracted heart tissue, the fatty acids were converted into fatty acid methyl esters (FAME) by the method of methanolic HCl. The FAME was separated and analyzed using a gas chromatography-mass spectrometry (GC-MS QP2010; Shimadzu Scientific Instruments) equipped with a 30 m fused-silica capillary column (30 m × 0.32 mm i.d., 0.25 *μ*m film thickness; Restek). The GC conditions were as follows: initial temperature of 130°C, 3°C min^−1^ to 240°C, injector and detector temperatures were 250°C, the column flow was 3.0 mL·min^−1^, the split ratio was 1 : 100, and 1 *μ*L injection volume was used. Fatty acid (FA) peaks were identified by using FAME standards (The Food Industry 37 FAME mix, 35077 Restek) and expressed as the percentage of total fatty acids. An overview of the identified monounsaturated, polyunsaturated, and saturated fatty acids is listed in Supplementary Table 1.

### 2.8. Determination of Oxysterols by LC-MS/MS in Heart Tissue

Oxysterols were isolated from the lipid extracts, according to the Bligh and Dyer method [19], by the solid-phase extraction (SPE) [20]. Following the isolation, oxysterols were then analyzed by LC-MS/MS after the derivatization with picolinic acid.

The MS method for the individual picolinic acid-oxysterol standard was analyzed in the “scan mode” of the single quadrupole mass spectrometer to determine if the oxysterols were derivatized to the compounds and fragmentation patterns described by Honda et al. [21]. The chromatographic separation of the picolinyl derivatives of oxysterols was carried out on the Shimadzu LC system, and the samples were analyzed and detected using the ESI-MS/MS detector in a positive mode. Hypersil GOLD C18 column (15 cm × 2.1 mm, 3 *μ*m particle size Thermo Electron) was used. Column oven temperature was maintained at 40°C, and the pump flow rate was maintained at 0.3 mL/min. The mobile phase was implemented in a gradient with 100% acetonitrile with 0.1% acetic acid (mobile phase A) in pump A and 100% water with 0.1% acetic acid in pump B (mobile phase B). The gradient program began with 80% mobile phase B for 2 min and was thereafter ramped to % 90 mobile phase B over a 28 min period and hold for 2 min, then returned to initial conditions.

The LC-MS/MS system consisted of an API 4000 QTrap triple quadrupole mass spectrometer (Applied Biosystems) through a TurboVTM ESI source and a Shimadzu UFLC system in which oxysterols 4*β*-hydroxycholesterol, 7-ketocholesterol, 25-hydroxycholesterol, and 27-hydroxycholesterol were analyzed.

### 2.9. Immunoblot Analysis

100 mg of heart tissues was homogenized in RIPA buffer (Cell Signaling) by using an Ultraturrax homogenizator at 15.000 rpm for 30 seconds and centrifuged at 15.000*g* for 20 minutes. The protein concentrations of the supernatants were determined by the Lowry method. 30 *μ*g of protein samples was separated with 10–12% SDS-PAGE gels and transferred to nitrocellulose or PVDF membranes. Membranes were probed with primary antibodies against SREBP1c and LXR*α* (Abcam). Following the use of HRP-conjugated secondary antibodies and chemiluminescence kit (Cell Signaling), blots were quantified and normalized with *β*-actin by densitometry using Image J software.

### 2.10. Gene Expression Analysis

100 mg of heart tissues was homogenized, and total RNAs were isolated with the RNA Midi Kit (QIAGEN) followed by reverse transcription using the Transcriptor High Fidelity cDNA Synthesis kit (ROCHE). Quantitative reverse transcriptase PCR was applied to cDNA with using the QuantiTect PCR Sybr Green kit (QIAGEN) and Rotor Gene Q-RT PCR system (QIAGEN). The threshold cycle (CT) was determined, and the relative gene expression subsequently was calculated as follows: fold change = 2^−Δ(ΔCT)^, where ΔCT = CT − CT target housekeeping (*β*-actin) and Δ(ΔCT) = ΔCT − CT-treated control. The sequences of primers used to detect the expression of rabbit transcripts are listed in Supplementary Table 2.

### 2.11. Statistical Analysis

Statistical analysis was performed using Prism 4 (Graph-Pad) software. For the determination of statistical significances of differences, one-way ANOVA was performed followed by multiple comparisons using Student's *t*-test. A *P* value less than 0.05 has been accepted to be statistically significant.

## 3. Results

### 3.1. Serum Cholesterol and *α*-Tocopherol Levels

To reveal the effect of a 2% cholesterol diet for 8 weeks on lipid and oxysterol metabolism in the heart tissue, we have established a well-known hypercholesterolemic rabbit model. In order to ensure our *in vivo* hypercholesterolemic model and supplementation of *α*-tocopherol, firstly, we measured cholesterol and *α*-tocopherol levels in serum. We have found an approximately 40-fold increase in serum cholesterol of 2% cholesterol-fed rabbits and a 10-fold increase of *α*-tocopherol in serum by its supplementation (Table 1) which supports our previous results [17, 22, 23].

### 3.2. Alpha-Tocopherol Levels and Oxidative Status in Heart Tissue

**Figure 1:**
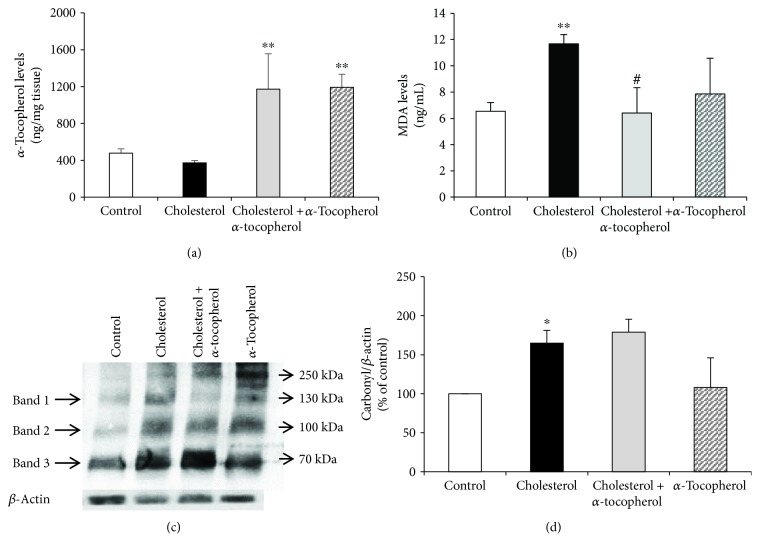
Alpha-tocopherol levels and oxidative status in heart tissue. Left ventricles of heart tissue were harvested, and *α*-tocopherol (a) and MDA levels (b) were quantified by LC-MS/MS. Protein carbonyl content was analyzed by Oxyblot following densitometric analysis of three different protein bands. Relative ratios were quantified and normalized using *β*-actin (c). Data are expressed as the mean ± SD. ^∗,∗∗^ *p* < 0.05 and 0.01 versus the control group; ^#^ *p* < 0.05 versus the cholesterol group (*n* = 3).

To determine the *α*-tocopherol content and the oxidation status of lipids and proteins, we examined *α*-tocopherol and MDA levels, together with the protein carbonyl formation, by LC-MS/MS and Oxyblot, respectively. As expected, increased levels of *α*-tocopherol were observed in heart tissues of cholesterol + *α*-tocopherol and *α*-tocopherol rabbits compared to control (Figure 1(a)). In the scope of lipid oxidation, the cholesterol group demonstrated an increase of MDA levels which was decreased to control levels by *α*-tocopherol supplementation (Figure 1(b)). Additionally, as shown in Figure 1(c), we found that a high cholesterol diet also induces protein carbonyl formation compared to control followed by no significant change in the cholesterol + *α*-tocopherol group compared to cholesterol.

### 3.3. Light Microscopy Examination of Heart Tissue

**Figure 2:**
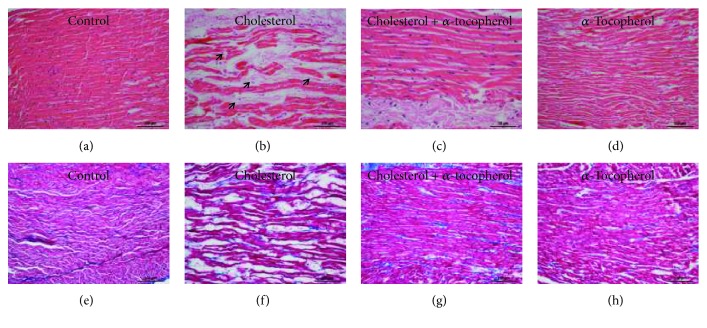
Light microscopic images of the myocardium from experimental animals. Fixed left ventricle of heart tissues in 10% buffered formaldehyde for 4 h dehydrated and incubated in xylol for 1 h twice, embedded in paraffin. 5 *μ*m thick sections were stained with hemotoxylene eosin (a–d) and Masson's trichrome (e–h) before microscopic examination. Representative H&E-stained sections from the control (a), cholesterol (b), cholesterol + *α*-tocopherol (c), and *α*-tocopherol (d) groups. A significant number of cardiomyocytes exhibited myofibrillar loss (arrows) in cholesterol group rabbits (b). Masson's trichrome-stained sections from the control (e), cholesterol (f), cholesterol + *α*-tocopherol (g), and *α*-tocopherol (h) groups show fibrotic remodeling of the left ventricle. No difference of fibrosis levels was observed between all groups of rabbits. Photographs taken at 200x magnification (scale bar = 100 *μ*m).

To evaluate the morphological features of the heart, hematoxylin-eosin (Figures 1(a)–1(d)) and Masson's trichrome (Figures 2(e)–2(h)) stainings were performed. While the control and *α*-tocopherol groups had normal myocardium morphology (Figures 2(a) and 2(d)), cholesterol-fed rabbits were indicated with heart tissue damage as increased myofibrillar loss which was reduced in animals supplemented *α*-tocopherol (Figures 2(b) and 2(c)). Additionally, Masson's trichrome-stained sections visualized with light microscopy showed no difference in fibrosis levels between the control (Figure 2(e)), cholesterol (Figure 2(f)), cholesterol + *α*-tocopherol (Figure 2(g)), and *α*-tocopherol (Figure 2(h)) groups of rabbits.

### 3.4. Free Fatty Acid Profiling following 2% Cholesterol Diet and *α*-Tocopherol Supplementation in Heart Tissue

**Figure 3:**
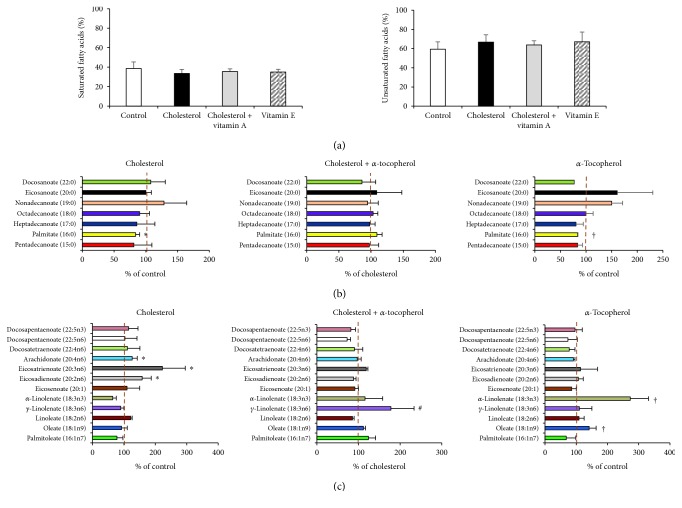
Fatty acid profile following 2% cholesterol diet and *α*-tocopherol supplementation. Left ventricles of heart tissue were harvested, and fatty acid content was analyzed by GC-MS. Levels of saturated fatty acids (SFAs) and unsaturated fatty acids (UFAs) (a). Complete profiles of SFAs (b) and UFAs (c). Data are expressed as the mean ± SD. ^∗^ *p* < 0.05 and ^†^ *p* < 0.05 versus the control group; ^#^ *p* < 0.05 versus the cholesterol group (*n* = 3).

Following the confirmation of our *in vivo* model, we have identified the alterations of free fatty acid composition in heart tissue by the GC-MS method. As shown in Figure 3(a), neither the 2% cholesterol diet nor *α*-tocopherol supplementation had any significant effect on total levels of saturated fatty acid (SFA) and unsaturated fatty acid (UFA). However, by evaluating the distribution of the different SFAs in the heart, we found a significant decrease of palmitate levels (16 : 0) in both the cholesterol and the *α*-tocopherol groups compared to control while no significant effect of *α*-tocopherol was observed in hypercholesterolemic rabbits (Figure 3(b)).

Moreover, at the scope of UFA distribution, increased levels of eicosadienoate (20:2n6), eicosatrienoate (20:3n6), and arachidonate (20:4n6) were found in the cholesterol group compared to control. Accumulation of oleate (18:1n9) and *α*-linolenate (18:3n3) was also observed in the *α*-tocopherol group compared to control. Interestingly, we found that *α*-tocopherol supplementation, in cholesterol-fed rabbits, showed its effect by increasing *γ*-linolenate (18:3n6) levels in the heart tissue (Figure 3(c)).

### 3.5. Oxysterol Profiling in Heart following High Cholesterol Diet and *α*-Tocopherol Supplementation

**Figure 4:**
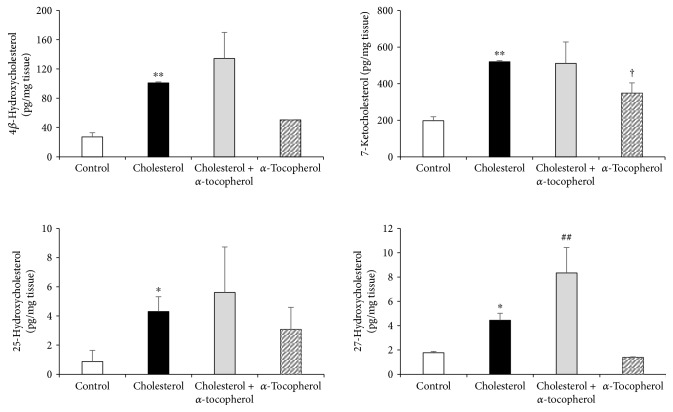
Effect of 2% of cholesterol diet and *α*-tocopherol supplementation on heart 4*β*-hydroxycholesterol, 7-ketocholesterol, 25-hydroxycholesterol, and 27-hydroxycholesterol concentrations. Oxysterol levels were quantified by LC-MS/MS in left ventricles of heart tissue. Data are expressed as the mean ± S.D. ^∗,∗∗^ *p* < 0.05 and 0.01 and ^†^ *p* < 0.05 versus the control group; ^##^ *p* < 0.01 versus the cholesterol group (*n* = 3).

Oxysterols occur enzymatically by side chain hydroxylation of cholesterol and were nonenzymatically formed by the attack of reactive oxygen species to the C-7 sterol ring of cholesterol [24]. Under induced inflammatory and oxidative stress conditions, excessive free cholesterol levels are prone to autoxidation to oxysterols which were identified as a major risk factor for cardiovascular disease development [25]. As shown in Figure 4, our LC-MS/MS findings from the heart tissue revealed that both 4*β*-, 25-, and 27-hydroxycholesterol and 7-ketocholesterol levels were increased significantly in the cholesterol group compared to control. Levels of 27-hydroxycholesterol also continued to increase significantly in the cholesterol + *α*-tocopherol group compared to cholesterol. This pattern was not observed in other oxysterols, such as 4*β*-hydroxycholesterol, 25-hydroxycholesterol, and 7-ketocholesterol. Additionally, *α*-tocopherol supplementation in normocholesterolemic rabbits showed its effect by increasing 7-ketocholesterol levels compared to control.

### 3.6. Expression of Fatty Acid and Cholesterol Metabolism-Related Parameters

**Figure 5:**
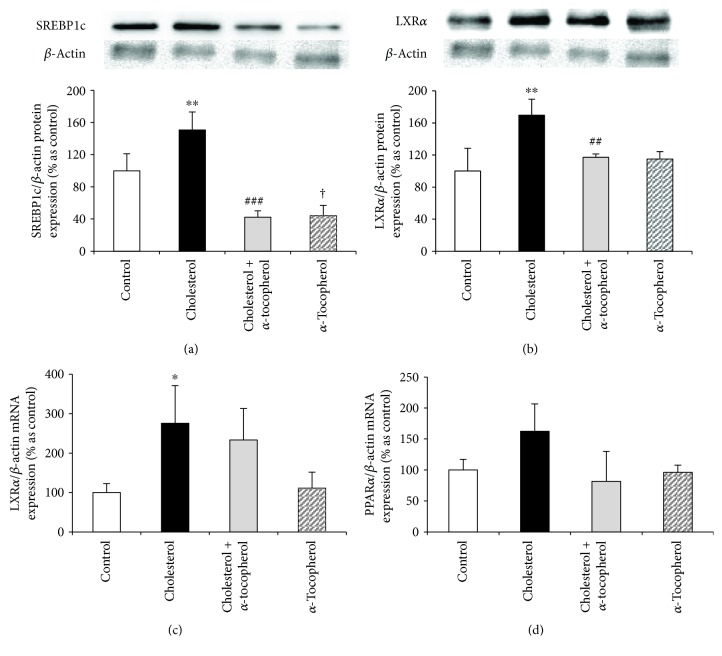
Expression of fatty acid and cholesterol metabolism-related parameters. Protein expressions in heart tissue of each animal were measured by Western blotting with densitometric analysis of protein bands, and relative ratios were quantified and normalized relative to *β*-actin against SREBP1c (a) and LXR*α* (b) antibodies. mRNA expressions of each animal were measured by RT-PCR and normalized to *β*-actin. Relative mRNA expressions of LXR*α* (c) and PPAR*α* (d). Data are expressed as mean ± S.D. ^∗,∗∗^ *p* < 0.05 and 0.01 and ^†^ *p* < 0.05 versus the control group; ^##,###^ *p* < 0.01 and 0.001 versus the cholesterol group (*n* = 3).

Increased levels of certain fatty acids and oxysterols direct us to evaluate gene/protein expressions of fatty acid and cholesterol metabolism parameters. LXR*α* and PPAR*α* are two important transcription factors that are highly expressed in the heart and promote the genes responsible for lipid metabolism, inflammation, and cholesterol efflux [7, 8]. In the view of our results and literature, we have investigated SREBP1c and LXR*α* protein expressions together with LXR*α* and PPAR*α* mRNA levels. In a translational manner, we have identified that both SREBP1c and LXR*α* expressions were significantly induced which were reduced to control levels by *α*-tocopherol supplementation (Figures 5(a) and 5(b)), although no band formation of cleaved SREBP1c was observed that was correlated with decreased palmitate levels by a high cholesterol diet. Similar to protein expression, the high cholesterol diet also induced the mRNA level of LXR*α* whereas *α*-tocopherol has no effect (Figure 5(c)). However, neither the high cholesterol diet nor *α*-tocopherol supplementation had any significant change on PPAR-*α* mRNA expression (Figure 5(d)). Taken together, LC-MS/MS, protein, and mRNA findings lead us to conclude that increased oxysterol levels might lead to the induction of LXR*α* signaling.

### 3.7. Alterations in Oxysterol Metabolism Effect Scavenger Receptor Expression and Reverse Cholesterol Transport

**Figure 6:**
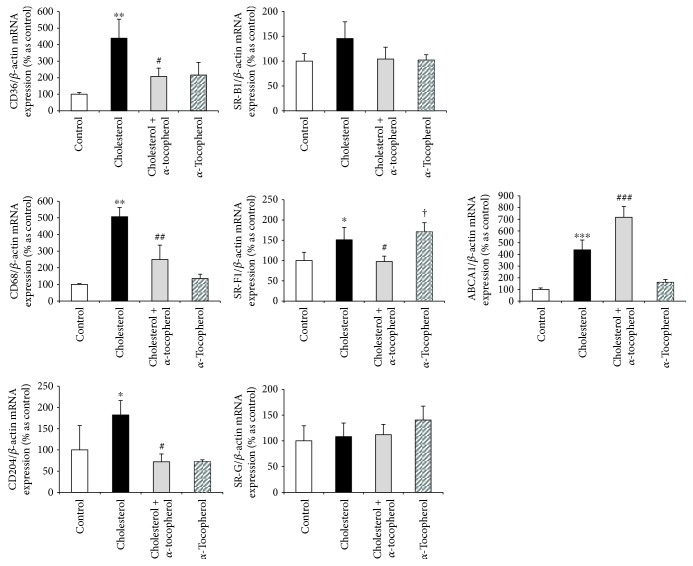
Alterations in oxysterol metabolism effect scavenger receptor expression and reverse cholesterol transport. mRNA expressions in heart tissue of each animal were measured by RT-PCR and normalized to *β*-actin. Relative mRNA expressions of CD36, CD68, CD204, SR-B1, SR-F1, SR-G, and ABCA1. Data are expressed as the mean ± SD. ^∗,∗∗,∗∗∗^ *p* < 0.05, 0.01, and 0.001 and ^†^ *p* < 0.05 versus the control group; ^#,##,###^ *p* < 0.05, 0.01, and 0.001 versus the cholesterol group (*n* = 3).

Although the involvement of scavenger receptors and reverse cholesterol transport is well documented in CVDs, the mechanistic link between oxysterols and SRs, following a high cholesterol diet, has not yet been fully identified. As shown in Figure 6, mRNA expressions of various scavenger receptors, such as CD36, CD68, CD204 (SR-A), and SR-F1, were induced by the high cholesterol diet while *α*-tocopherol supplementation significantly inhibited this induction. We also observed that *α*-tocopherol supplementation, in normocholesterolemic rabbits, showed its effect by inducing the mRNA levels of class F scavenger receptor (SR-F1).

In order to evaluate the modulations in the reverse cholesterol transport system, we evaluated the mRNA expression of ABCA1 by qPCR. Our findings revealed an approximately 5-fold increase in mRNA expression of ABCA1 in the cholesterol-fed group which was continued to increase significantly by *α*-tocopherol supplementation in the cholesterol + *α*-tocopherol group (Figure 6). These results were also parallel to 27-hydroxycholesterol levels in our *in vivo* model and led us to hypothesize a boosting effect of *α*-tocopherol in a reverse cholesterol transport through 27-hydroxycholesterol induction.

## 4. Discussion

With the highest morbidity and mortality rates, CVDs are the leading death cause, globally. According to the WHO report, approximately 17.5 million people, which equals to 31% of all deaths, died from CVDs in 2012 [26]. Epidemiological studies identified elevated LDL cholesterol levels, even the absence of other risk factors might enhance the progression of CVD [27]. However, there is little information in the literature about the alterations of fatty acid and oxysterol profiles and scavenger receptors following a high cholesterol diet and *α*-tocopherol supplementation. Based on our hypercholesterolemic rabbit model, we showed that the 2% cholesterol diet and *α*-tocopherol supplementation for 8 weeks might be associated with changed free fatty acid and oxysterol compositions, proteins related to lipid metabolism, and scavenger receptors in parallel to histological alterations.

Our high cholesterol-fed rabbits showed significantly increased serum cholesterol levels compared to controls. Serum *α*-tocopherol levels were also enhanced in *α*-tocopherol-supplemented rabbits. *α-*Tocopherol is a beneficial nutrient transported in plasma lipoproteins because of its hydrophobic nature. The observation of increased serum *α*-tocopherol levels in the cholesterol group, compared to control, is mostly based on that *α*-tocopherol is a fat-soluble vitamin carried by LDL in the blood and a higher *α*-tocopherol uptake caused by the increased lipid uptake produced by the high cholesterol diet. Despite the increase of *α*-tocopherol levels in serum samples of the cholesterol group, we did not observe any change in heart tissue samples of the same animals. These results prove that tissue levels of *α*-tocopherol increased mostly by daily injections and *α*-tocopherol-mediated regulations can only be hypothesized in the cholesterol + *α*-tocopherol and *α*-tocopherol groups.

The use of MDA and protein carbonyls as a parameter of lipid and protein oxidation is accepted in patients during coronary heart surgery [28]. In the present study, detection of high MDA levels and protein carbonyl formation in the heart tissue reflects the effect of the high cholesterol diet on oxidized lipid and protein accumulation *in vivo*. We also demonstrated that *α*-tocopherol supplementation had the capacity of decreasing high cholesterol diet-induced MDA levels with no influence on protein carbonylation. Altogether, cholesterol, *α*-tocopherol, and oxidative stress results indicate a positive correlation between hypercholesterolemia and oxidative stress, which supports our previous results [17, 22, 23] and also is in agreement with the literature [29, 30].

Recent studies have classified the progression of cardiomyopathy into four classes on the basis of pathology. While class I is characterized by cardiomyocyte alterations, class II contains fibrotic remodeling, class III exhibits fibrosis and cardiomyocyte damage, and class IV consists the accumulation of noncollagenous material or inflammatory response with or without cardiomyocyte alterations [31]. Our light microscopy findings from the cholesterol-fed group exhibited the loss of myofibrils, without effecting fibrosis, which suggests the presence of cardiomyopathy with class I characteristics. Moreover, cholesterol-fed rabbits undergoing *α*-tocopherol supplementation showed reduced alteration in the morphology of the myocardium.

Although the oxidation of fatty acids is the major energy source for contractility, in regard to limited de novo synthesis capacity, cardiomyocytes rely heavily on the uptake of fatty acids in the form of nonesterified (free) or lipoproteins [32]. To date, three major transport proteins are identified for the uptake of circulating fatty acids: CD36 (also known as fatty acid translocase (FAT)), fatty acid-binding protein (FABP), and fatty acid transport protein (FATP) [33]. To examine the effect of SFAs versus UFAs in primary cardiomyocytes, Vries et al. have identified C16:0 (palmitate) or C18:0 (stearate) has a capacity to induce apoptosis, while C16:1 (palmitoleate) or cis-C18:1 (oleate) has no effect on cell viability [34]. Our GC-MS findings lead us to conclude, despite the unchanged level of total UFAs, eicosadienoate (20:2n6), eicosatrienoate (20:3n6), and arachidonate (20:4n6) levels were increased in the cholesterol group. *γ*-Linolenate (18:3n6) levels were also increased in the cholesterol + *α*-tocopherol group compared to cholesterol (Figure 3). Since the accumulation of eicosatrienoate (20:3n6) and arachidonate (20:4n6) can induce inflammation [35, 36], this might enlighten our previous results (using same tissue samples) which determines an induction of inflammatory cytokines (despite the absence of inflammatory infiltration histologically) in cholesterol-fed rabbits (unpublished data). However, the decrease of palmitate levels in the cholesterol and *α*-tocopherol groups, without effecting oxysterol and scavenger receptor expressions, might be based on the increased energy consumption. Further studies are needed to reveal this hypothesis.

Oxygenated derivatives of cholesterol (oxysterols) are biologically active molecules and, due to their lipophilic composition, easily penetrate from macrophages into the surrounding cells, including cardiac cells. Increased ROS levels in the macrophage are implied as the major source of oxysterol production in all tissues [2]. Association of cardiomyocyte oxysterols with CVD development is reported as an important factor through the regulation of cell hypertrophy and death [25]. Additionally, the involvement of oxysterols has been identified either in the plasma samples of coronary artery bypass grafted patients [37, 38] or in those of cardiac catheterized patients [39]. A variety of oxysterols, including 4*β*-hydroxycholesterol, 7-ketocholesterol, 25-hydroxycholesterol, and 27-hydroxycholesterol is also investigated by LC-MS/MS experiments. As shown in Figure 4, the high cholesterol diet for 8 weeks had the capacity to enhance all four oxysterol levels. In contrast to the other oxysterols, 27-hydroxycholesterol continued to increase significantly in hypercholesterolemic animals supplemented with *α*-tocopherol. The effect of *α*-tocopherol supplementation in normocholesterolemic animals was also tested, and only 7-ketocholesterol was found to increase in the left ventricle of the heart tissue. Thus far, our results show that the boosting role of *α*-tocopherol on 7-ketocholesterol in normocholesterolemic animals has no significant effect on scavenger receptor levels in the heart tissue.

Following the delivery of cholesterol into the cell, it is transported either to ER, facilitating the sequestration of SREBPs that reduces cholesterol synthesis and uptake, or to the plasma membrane, enhancing cholesterol efflux by ABCA1. LXR*α* is a nuclear receptor which is highly expressed in macrophages. Following the activation by a number of oxysterols, such as 25- and 27-hydroxycholesterol, LXR*α* acts as a transcription factor and enhances the expression of genes involved in cholesterol homeostasis, fatty acid metabolism, and inflammation. It is clear that 27-hydroxycholesterol-mediated LXR*α* activation enhances cholesterol efflux via ATP-binding cassette transporters, such as ABCA1 and ABCG1, and reduces the expression of proinflammatory cytokines [1]. In this regard, a number of studies have also determined oxysterol-mediated LXR*α* activation as a crucial step in reverse-cholesterol transport and foam cell formation by inducing ABCA1 levels [40, 41]. Oxysterol-mediated LXR*α* activation might also enhance the transcription of sterol regulatory element-binding protein 1c (SREBP1c) that stimulates lipogenesis [8]. Besides the LXR*α*, oxysterols have been shown to accelerate SREBP sequestration by interacting with Insig-1/2 [1]. Similar to the protein level, mRNA expression of LXR*α* was induced by the high cholesterol diet, without any change in PPAR*α* mRNA. Moreover, despite the significant increase of the uncleaved form of SREBP1c, no band formation of cleaved SREBP1c was observed, which is correlated with a decrease in palmitate levels and led us to hypothesize the downregulation of lipogenesis by the high cholesterol diet (Figure 5).

Our oxysterol and LXR data suggested to us a theory involving SRs. SRs are crucial transporters, together with lipoprotein receptors, in the delivery of exogenous cholesterol to endocytic pathways. CD36 is one of the most studied SRs, which binds a number of ligands including long-chain fatty acids, apoptotic cells, and OxLDL, in a high-fat diet, inflammation, or oxidative stress. [42]. Leonarduzzi et al. have observed a marked increase in both synthesis and expression of CD36 on human U937 promonocytic cells by using an oxysterol mixture, which contains major oxysterols of dietary origin but not 27-hydroxycholesterol [43]. In another study, the oxysterol mixture (contains high rate of 27-hydroxycholesterol compared to Leonarduzzi et al.) reduced the expression of CD36 and CD204 SRs, while upregulating LXR*α* and ABCA1 levels in the stimulation of macrophage polarization toward the M2 phenotype [44]. Moreover, in contrast to 25-hydroxycholesterol, 7-ketocholesterol treatment has been described to promote THP-1 differentiation, by increasing CD11b, CD36, and CD68 expressions, and leads to foam cell formation following the exposure of oxLDL [45]. In our study, the marked increase of various scavenger receptors, including CD36, CD68, CD204, and SR-F1, was observed in heart tissues of rabbits fed with the high cholesterol diet and these increases were completely inhibited by *α*-tocopherol supplementation. A similar increase in cholesterol rabbits was also obtained in ABCA1 levels which was differently continued to increase with *α*-tocopherol, similar to 27-hydroxycholesterol.

PPAR*α* is another lipid-activated transcription factor and known to modulate lipid metabolism in the progression of atherosclerosis. Scavenger receptor class B type I (SR-BI), responsible for HDL-mediated cholesterol efflux, is shown to regulate by both PPAR-*α* ligands in human macrophages and atherosclerotic lesions of apoE knockout mice [46]. In our study, we did not observe any significant effect of the high cholesterol diet and *α*-tocopherol supplementation either in PPAR*α* or in SR-B1 mRNA levels which proved ABCA1 was more effective in RCT in the heart tissue of our model.

Further studies have shown that 7-ketocholesterol-related changes in various gene-involved different pathways, such as inflammation and lipid homeostasis, might be regulated by vitamin E [47]. In a related study, 7-ketocholesterol-mediated activation of oxiapoptophagy, a mixed form of cell death that contains the features of oxidation, apoptosis, and autophagy, has been reported to be downregulated by *α*-tocopherol [48]. Additionally, 7-ketocholesterol-induced autophagic death of human smooth muscle cells has also been reported [49]. In our unpublished results, we have determined that excessively induced ER stress triggers autophagic activity in the same tissue samples of cholesterol-fed rabbits, which results in the loss of cardiomyocytes by autophagic cell death.

This study shows that the high cholesterol diet did not change total saturated/unsaturated fatty acid levels but increased oxysterols, particularly 7-ketocholesterol, stimulated various SR expressions, such as CD36, CD68, CD204, and SR-F1. At the same time, 25-hydroxycholesterol and 27-hydroxycholesterol-mediated LXR activation upregulated ABCA1 levels. *α*-Tocopherol supplementation, together with the high cholesterol diet, showed its effect by decreasing SR expressions in a transcriptional manner, while continuing to boost reverse transport of excess cholesterol through 27-hydroxycholesterol and ABCA1 levels without effecting LXR*α* expression. *α*-Tocopherol shows its beneficial effects on various diseases such as atherosclerosis, coronary heart diseases, neurodegenerative diseases, and cancer [50, 51]. According to Poirier et al., *α*-tocopherol has the capacity to increase 27-hydroxycholesterol synthesis following the induction in oxysterol-generating enzyme, hepatic 27-hydroxylase (CYP27A1), expression [52]. Based on the literature and our previous results in aorta tissue [17], we hypothesized that *α*-tocopherol supplementation might show its beneficial effect by increasing ABCA1 expression through 27-hydroxycholesterol in hypercholesterolemic conditions.

In conclusion, this study presents a comprehensive data of the alterations in fatty acids, oxysterols, and SR composition and their implication with histological modifications of the heart tissue in cholesterol-fed rabbits. Future studies are also needed to identify the biological association of the identified changes in the lipid profile, as well as the effect of *α*-tocopherol supplementation. In addition to the present study, new findings in the progression of lipid metabolism and SR alterations will not only increase our understanding but also lead the development of further theories and therapeutic strategies in the field of hypercholesterolemia-mediated heart diseases.

## Figures and Tables

**Table 1:** Effect of 2% cholesterol diet and *α*-tocopherol supplementation on serum cholesterol and *α*-tocopherol levels following eight weeks.

	Serum cholesterol levels (mg/dL)	Serum *α*-tocopherol levels (*μ*g/mL)
Control	52.0 ± 9.8	2.8 ± 1.3
Cholesterol	2027.2 ± 860.4^∗∗∗^	24.5 ± 3.4^∗∗∗^
Cholesterol + *α*-tocopherol	2341.4 ± 552.5^∗∗∗^	21.1 ± 4.7^∗∗∗^
*α*-Tocopherol	60.2 ± 23.9	17.3 ± 10.3^∗∗∗^

Data are expressed as the mean ± SD. ^∗∗∗^ *p* < 0.001 versus the control group (*n* = 5).

## Supplementary Materials

Supplementary Materials Supplementary material contains an overview of the identified fatty acids and the sequences of primers to detect the expression of rabbit transcripts. Supplementary Table 1: identified fatty acid profile which contains trivial and the IUPAC names together with short-hand nomenclature. Supplementary Table 2: primer sequences used for the quantitative real-time PCR experiments.
