# Supplementary material for: High Cholesterol Diet-Induced Changes in Oxysterol and Scavenger Receptor Levels in Heart Tissue
Source: Oxid Med Cell Longev. 2018 Jun 13;2018:8520746. doi: 10.1155/2018/8520746 (PMC6020519; doi:10.1155/2018/8520746)
Supplement: Supplementary Materials — Supplementary material contains an overview of the identified fatty acids and the sequences of primers to detect the expression of rabbit transcripts. Supplementary Table 1: identified fatty acid profile which contains trivial and the IUPAC names together with short-hand nomenclature. Supplementary Table 2: primer sequences used for the quantitative real-time PCR experiments. [file 8520746.f1.pdf]

Supplemental Table 1. Identified fatty acid profile which contains trivial and the IUPAC names together with short-hand nomenclature.

Abbreviation	Trivial name	International Union of Pure and Applied Chemistry name
C15:0	Pentadecylic acid	Pentadecanoic acid
C16:0	Palmitic acid	Hexadecanoic acid
C16:1n-7	Palmitoleic acid	(9Z)-Hexadec-9-enoic acid
C17:0	Margaric acid	Heptadecanoic acid
C18:0	Stearic acid	Octadecanoic acid
C18:1n-9	Oleic acid	(9Z)-Octadec-9-enoic acid
C18:2n-6	Linoleic acid	(9Z,12Z)-Octadeca-9,12-dienoic acid
C18:3n-6	γ-linolenic acid	(6Z,9Z,12Z)-Octadeca-6,9,12-trienoic acid
C18:3n-3	α-linolenic acid	(9Z,12Z,15Z)-octadeca-9,12,15-trienoic acid
C19:0	Nonadecylic acid	Nonadecanoic acid
C20:0	Arachidic acid	Icosanoic acid
C20:1n-9	Gondoic acid	(11Z)-Eicos-11-enoic acid
C20:2n-6	Eicosadienoic acid	(11Z,14Z)-Icosa-11,14-dienoic acid
C20:3n-6	Dihomo-γ-linolenic acid	(8Z,11Z,14Z)-Icosa-8,11,14-trienoic acid
C20:4n-6	Arachidonic acid	(5Z,8Z,11Z,14Z)-Icosa-5,8,11,14-tetraenoic acid
C22:0	Behenic acid	Docosanoic acid
C22:4n-6	Adrenic acid	(7Z,10Z,13Z,16Z)-Docosa-7,10,13,16-tetraenoic acid
C22:5n-6	Osbond acid	(4Z,7Z,10Z,13Z,16Z)-Docosa-4,7,10,13,16-pentaenoic acid
C22:5n-3	Docosapentaenoic acid	(7Z,10Z,13Z,16Z,19Z)-docosa-7,10,13,16,19-pentaenoic acid

Supplemental Table 2. Primer sequences used for the quantitative real-time PCR experiments.

Gene	5’ Primer	3’ Primer
SR-B2 (CD36)	CCTCCTTGGCCTGGTAGAAA	TGTGCTACAGAGGAGAGTGC
SR-D1 (CD68)	TCCAGTTGAGAGTCGGCATT	GGAGTGGCAGTTGTAGGTCT
SR-A (CD204)	TGGACCAAAAGGCCAAAAGG	GGGACCAGTACCTTGTCCAA
SR-B1	CAACGACAATGACACCGTGT	TCGTAGCCCCACATGATCTC
SR-F1	GGCTCAGGATAGGTCACTCC	CGCTGGAACTGAACTGCTTT
SR-G	CCAAGTACACAGAGGAGGCA	CTAGCTGCTTCTGGTTCTGC
ABCA1	ACTTGCTCAGCGGGATGGAT	AGGCTGAACACCAAGGCGAT
β-actin	ACGAAACCTAACGGCGCAGA	TTTGGGGATGCTCGCTCCAA
